# Multiple Recurrences of Trigeminal Neuralgia Caused by Deformation of the Trigeminal Nerve

**DOI:** 10.7759/cureus.6433

**Published:** 2019-12-20

**Authors:** Zaid Aljuboori, Haring J Nauta

**Affiliations:** 1 Neurosurgery, University of Louisville School of Medicine, Louisville, USA

**Keywords:** trigeminal neuralgia, pain, face, nerve, padding, decompression.

## Abstract

Microvascular decompression (MVD) is frequently used for the treatment of trigeminal neuralgia (TN). We present a case of TN with multiple recurrences despite different interventions. A 50-year-old patient presented with a five-year history of left TN. He initially had an MVD with Teflon padding that eliminated his pain for a year. When pain recurred, he went on to stereotactic radiosurgery, which did not help. He then had two percutaneous retrogasserian glycerol injections, the first one relieved pain for a year and the second only six months. After the second recurrence, we repeated the magnetic resonance imaging (MRI) of the brain, and it showed kinking of the nerve with the padding in place. We decided to re-explore the nerve based on the MRI findings. Intraoperatively, we observed the Teflon padding had become adherent to the petrous bone which caused deformation of the nerve. We did adhesiolysis with debulking of the padding, following which the nerve appeared more relaxed. Postoperatively, the patient had immediate resolution of his pain. At eight-month follow-up, the patient remained pain-free. Multiple factors can be involved in recurrence of TN after MVD. In this case, the size of the padding, continued distortion from the offending artery in addition to scarring from radiosurgery may have contributed to the deformation of the nerve and the recurrence of symptoms. A new MRI can be beneficial when the neuralgia symptoms recur in delayed fashion after successful MVD. Also, the use of a more compact padding material, like Gore-Tex, may cause less deformation of the nerve.

## Introduction

Trigeminal neuralgia (TN) is a paroxysmal, recurrent, lancinating, or electric shock-like pain [1]. The pain is usually localized to one or more of the three main divisions of the trigeminal nerve [2]. Microvascular decompression (MVD) is frequently used in the treatment of typical TN, with pain recurrence occurring in about 10%-30% of patients [3]. Multiple factors have been implicated in the delayed recurrence of symptoms, including new or additional vascular compression, formation of arachnoid adhesions or granuloma, and dislodgement of the padding [4]. There is no consensus as to the optimal treatment, some advocated repeat MVD, while others suggested less invasive procedures such as percutaneous radiofrequency ablation or glycerol injection (GI) [1-7]. Also, the use of stereotactic radiosurgery (SRS) in recurrent TN was investigated [3,5]. Furthermore, the literature is not clear as to the necessity for MRI of the brain after recurrence. We present a case of typical TN with multiple recurrences despite different interventions.

## Case presentation

**Figure 1:**
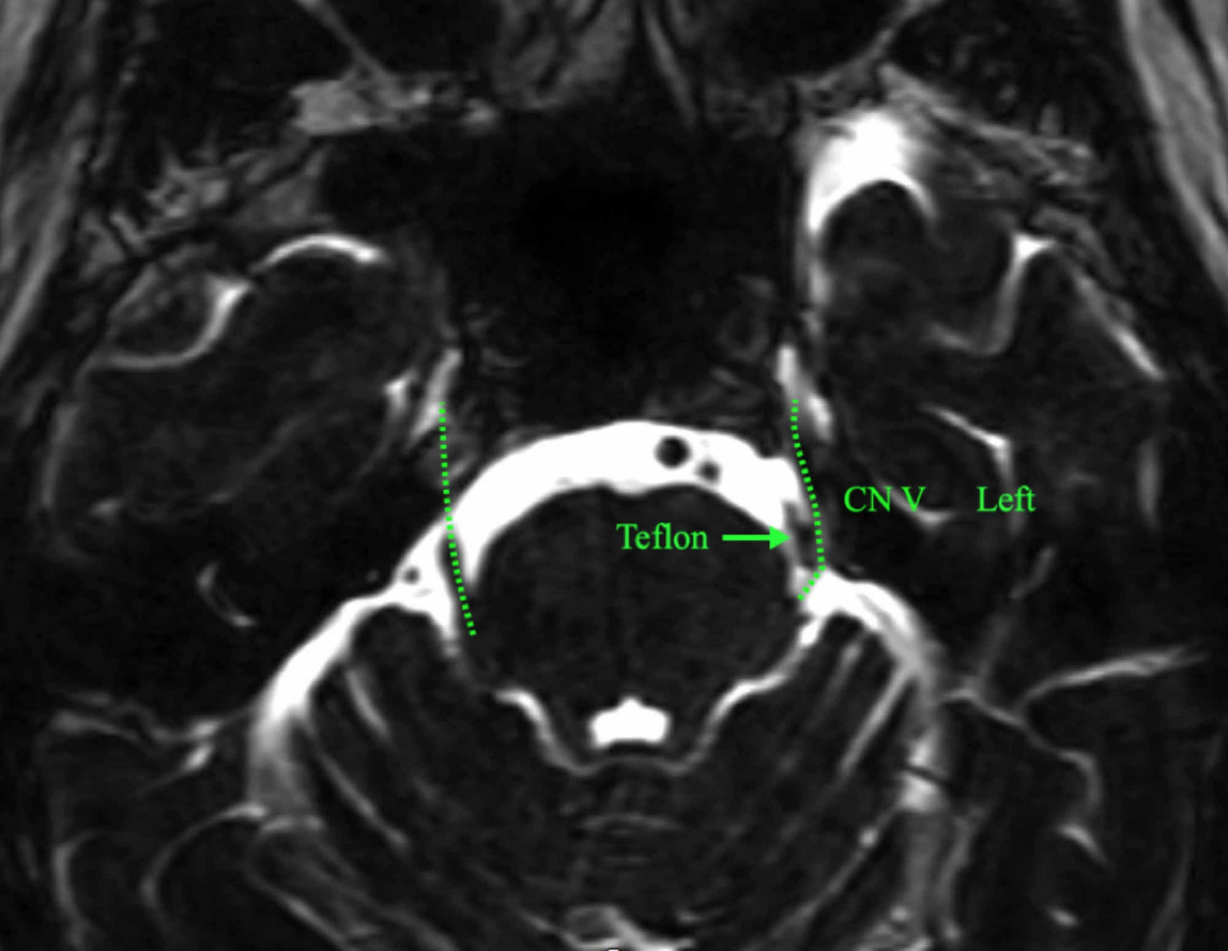
MRI of the brain shows posterior kinking of the left trigeminal nerve (CN V) with the padding seeming to be in place.

A 50-year-old man presented with a five-year history of left V2 and V3 TN, and the pain was frequent with multiple episodes on daily basis, sharp in nature with an electric shock-like sensation. There were multiple triggers including skin touch and chewing. His pain score was 5 according to the Barrow Neurological Institute (BNI) pain intensity score. He initially had an MVD with Teflon felt padding that eliminated his pain (BNI grade 0) for a year. Then pain recurred spontaneously, and it was similar in quality and intensity (BNI grade 5) to his initial pain. He then was treated with SRS with an 80 Gray in a single fraction that targeted the transitional zone, but there was no improvement. He then had two percutaneous retrogasserian GIs, the first relieved pain for a year and the second for only six months. We repeated his MRI scan (Fast Imaging Employing Steady-state Acquisition [FIESTA] protocol), and it showed a dramatic posterior kinking of the nerve with the padding apparently still in place (Figure 1).

 

**Figure 2:**
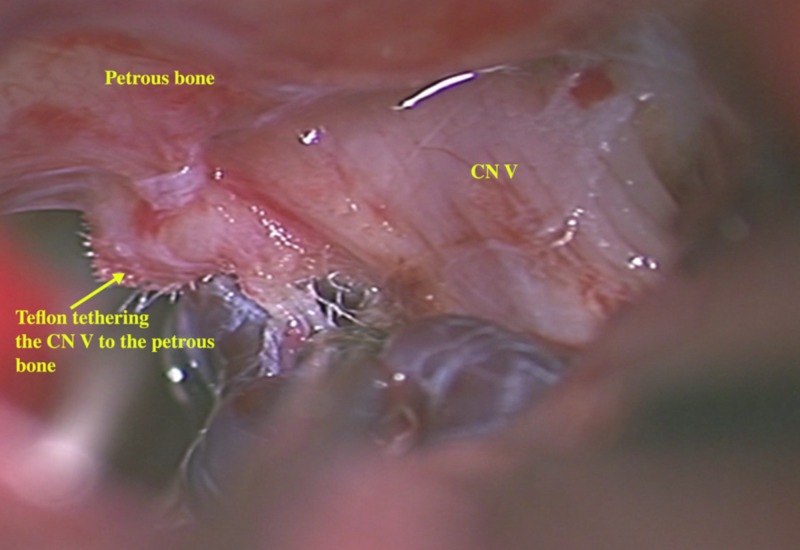
Intraoperative microscopic picture (50x) shows the left trigeminal nerve (CN V) kinked due to tethering of the Teflon pad to the petrous ridge, with the pad lies in between the nerve and the offending superior cerebellar artery.

**Figure 3:**
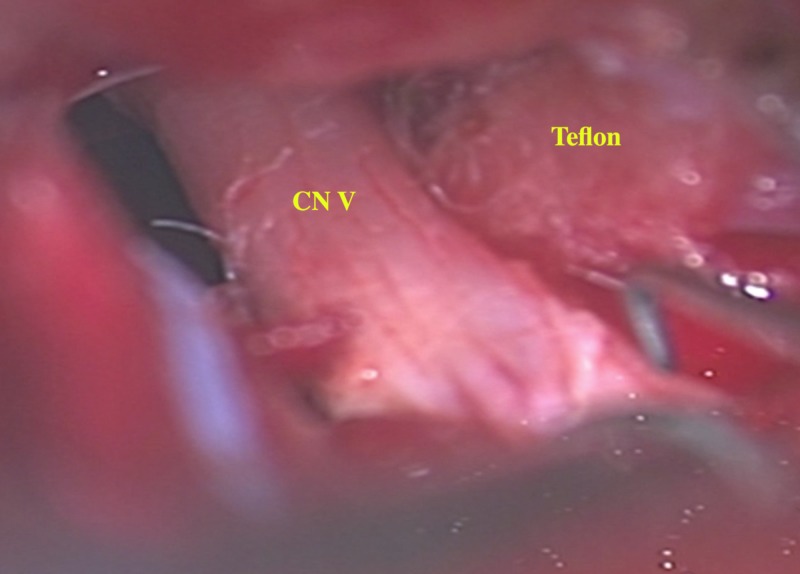
Intraoperative microscopic picture (50x) shows left trigeminal nerve (CN V) assuming a more natural course.

We considered repeat GI versus exploration, and following a discussion with the patient, decided that a re-exploration of the nerve was warranted based on the MRI findings showing the obvious nerve distortion possibly amenable to microsurgical correction. Intraoperatively, we observed that the Teflon padding was still in place between the nerve and the superior cerebellar artery (SCA) with both the nerve and the artery were severely to the pad. The caudal edge had become posteriorly displaced and scarred to the dura over petrous bone tethering and deforming the nerve (Figure 2). We did adhesiolysis with central debulking of the padding, following which the distorted course of the nerve appeared straighter and more relaxed (Figure 3). The SCA remained scarred away from the nerve. The brainstem auditory-evoked potentials remained stable.

Postoperatively, the patient reported immediate resolution of his pain with only mild residual numbness in the V2/V3 distribution unchanged from preop. Although most patients develop new or increased numbness after MVD, this was not evident in our case. This can be explained by the lack of significant manipulation of the nerve as the bulk of our microdissection was to release the adhesions between the Teflon pad and the petrous ridge, and debulking the pad itself. 

There was no hearing loss or facial weakness. At eight-month follow-up, the patient remained pain-free. 

## Discussion

MVD is frequently used in the treatment of typical TN after failure of initial medical management. It is the most successful option and is optimal for otherwise healthy patients. Longitudinal studies have shown that MVD provides an initial and long-term pain relief in more than 90% and 70% of patients, respectively. These results are higher than other treatment modalities. It also provides the highest long-term patient satisfaction [8-11]. Approximately one third of patients suffer from recurrence of TN five years after MVD. In 70% of such patients, recurrence was attributed to compression by an artery, vein or the Teflon pad itself, while in 30% no obvious cause was found [1,3,12].

The available treatment options for recurrent TN include redo MVD, percutaneous glycerol or radiofrequency rhizotomy, SRS, and percutaneous balloon compression [3,12,13].

In this case, after discussing the available treatment options with the patient, it was decided that SRS would be the next step in the treatment of the recurrent symptoms. This decision was reached by weighing the perceived risks of redo MVD versus the high safety profile of SRS, and acknowledged the lack of treatment guidelines for recurrent TN. In this case, transmitted distortion by the SCA through the bulk of the Teflon padding, in addition to scarring from radiosurgery, may have resulted in the deformation of the nerve, and the multiple recurrences of symptoms despite multiple subsequent interventions.

Our experience with this case indicates that a repeat MRI after the initial recurrence of symptoms is warranted as it can identify the position of the padding, and the offending artery. It will also show if deformation of the nerve has developed. In the case of nerve deformation or loss of the padding, a re-exploration is probably the best option. It may result in lower cumulative risk and cost than multiple less durable procedures to treat multiple recurrences of TN. Cheng et al. reported that redo MVD provided immediate pain relief in 80% of patients for a mean duration of two years, and partial pain relief in an additional 7% of patients. The complications were higher in redo MVD, hearing loss 5%, wound infection 2.5%, and cerebrospinal fluid leak 2.5% [1]. Raygor et al. reported that repeat SRS for the treatment of recurrent TN after an initial SRS resulted in long-term pain control in 33.3% of patients in comparison to 80% in repeat MVD group [3].

Jafree et al. reported that repeat MVD yielded the highest proportion of long-term pain relief at five years (66.7%) in patients with recurrent TN after initial MVD [13]. We suggest obtaining a new MRI when the neuralgia symptoms recur in delayed fashion. Also, the use of a more compact material for padding, like Gore-Tex in place of bulky Teflon felt, may be less prone to cause a deformation of the nerve.

## Conclusions

Recurrence of TN after a successful initial MVD can be attributed to different factors such as padding, deformation of the nerve due to adhesions, and continuous irritation by the offending vessel. A re-exploration can result in pain relief in the majority of patients.
